# Maximizing Engagement in Mobile Health Studies: Lessons Learned and Future Directions

**DOI:** 10.1016/j.rdc.2019.01.004

**Published:** 2019-05

**Authors:** Katie L. Druce, William G. Dixon, John McBeth

**Affiliations:** a Arthritis Research UK Centre for Epidemiology, University of Manchester, Manchester, UK; b NIHR Manchester Musculoskeletal Biomedical Research Unit, Central Manchester University Hospitals NHS Foundation Trust, Manchester, UK

**Keywords:** Epidemiology, mHealth, Methods, Remote monitoring, Rheumatic diseases, Patient reported outcomes

## Abstract

The widespread availability of smartphones, tablets, and smartwatches has led to exponential growth in the number of mobile health (mHealth) studies conducted. Although promising, the key challenge of all apps (both for research and nonresearch) is the high attrition rate of participants and users. Numerous factors have been identified as potentially influencing engagement, and it is important that researchers consider these and how best to overcome them within their studies. This article discusses lessons learned from attempting to maximize engagement in 2 successful UK mHealth studies—Cloudy with a Chance of Pain and Quality of Life, Sleep and Rheumatoid Arthritis.

## Key points

• The widespread availability of smartphones, tablets, and apps presents an exciting opportunity for epidemiologic research.
• Although promising, the key challenge of all apps (both for research and nonresearch) is the high attrition rate of participants and users.
• Any engagement strategies used should consider usability of technology, push or motivating factors, and the need for personal contact with study personnel (not just technology) and study support.
• Particular benefits to long-term engagement may occur through the use of real-time data monitoring and passive monitoring and by providing personalized study feedback.
• Future studies should consider adopting and advancing these approaches at an early stage of study design to maximize patient engagement.

## Introduction

### The Promise of Apps to Support Data Collection for Health Research

The widespread availability of smartphones, tablets, and smartwatches and common usage of apps,^1^, ^2^ particularly for health care monitoring,^3^, ^4^ present an exciting opportunity for epidemiologic research to reach and recruit high proportions of the population. Furthermore, using apps and other mobile health (mHealth) technology, such as wearables, researchers can now conduct frequent and repeated remote collection of self-report data, such as symptom reports, and objective assessments of biometrics (eg, heart rate), sleep, physical activity, and active tasks, such as walking tasks to determine gait and balance.^5^, ^6^, ^7^, ^8^ mHealth studies can thus increasingly provide data to investigate day-to-day patterns of disease and embed objective markers of symptom and disease factors through time more readily than traditional models (eg, registries), which tend to investigate change between disparate time points (eg, every 6–12 months).

### The Challenge of Maintaining Engagement

Although promising, a fundamental challenge for those seeking to conduct app studies is the seemingly ubiquitous phenomenon of rapid and substantial disengagement from apps^9^ for both research and nonresearch. For example, despite one of the most successful smartphone games of recent years, Pokémon Go (https://pokemongolive.com/en/) experienced a loss of one-third (15 million) of their daily active users within 1 month of the launch date.^10^ Estimates indicate that approximately 71% of app users across all industries (eg, media and entertainment, retail, lifestyle, and business) disengage within 90 days^11^; in health research studies, as few as approximately 10% to 25% of recruited participants have been shown engaged in studies by the end of data-entry protocols that collect data from once per week to 3 times a day, lasting between 1 week and 12 weeks.^5^, ^12^, ^13^

Among research studies, loss of engagement can have substantial impacts on the integrity of data collected within mHealth studies, creating issues, such as bias (if those who disengage are systematically different from those who do not), reduced data quality, and high rates of data missingness. Furthermore, lack of transparency in study design or reporting can lead to misinterpretation if it is not clear how many people entered and remained in a study and what the factors are that drive that engagement. Failure to address or prevent these threats may mean that mHealth studies produce incorrect conclusions about the existence, strength, or direction of associations between exposures and outcomes.^14^

For those seeking to overcome these challenges, the importance of maximizing participant engagement is paramount. Insights also may be obtained from reflecting on the design and engagement processes used in other successful mHealth studies.

### The Success of Apps

Two recently completed mHealth studies conducted within the Arthritis Research UK Centre for Epidemiology at the University of Manchester have had notable success with respect to recruiting and engaging participants for between 30 days and 12 months.

The first study, Cloudy with a Chance of Pain,^15^, ^16^ is a UK smartphone-based study that sought to examine the link between the weather and pain in people with chronic pain. Participants were recruited to the study using a variety of advertisements both in mainstream and social media. Importantly, participants had no contact with the research team prior to registration and instead accessed the study Web site, self-downloaded the app, and registered remotely. After registration, participants were asked to complete a baseline questionnaire and report symptoms once per day (estimated 1–2 minutes per entry) for 6 months (latterly extended to 12 months). Meanwhile, the smartphone’s Global Positioning System (GPS) reported hourly location, allowing linkage to local weather data from the Met Office (the UK national weather service). The authors demonstrated that approximately 1 in 7 participants were highly engaged for 6 months in a study investigating whether self-reported pain severity is associated with weather variables, completing full data entry on 89% of all possible days.^15^

The second study, Quality of Life, Sleep and Rheumatoid Arthritis (QUASAR), examined the relationship between sleep and quality of life among people with rheumatoid arthritis. Participants were recruited through advertisements disseminated by the UK National Rheumatoid Arthritis Society and displayed in several National Health Service secondary care clinics (or rheumatology offices) and primary care surgeries (or doctors’ offices). Potential participants completed an online screening questionnaire and were recruited directly by the study team during recruitment telephone calls. After completion of a baseline questionnaire, digital data collection comprised a morning daily sleep diary and symptom assessment (estimated 5–7 minutes of data entry) and an evening symptom assessment (estimated 1–2 minutes of data entry) for 30 days and a series of follow-up questionnaires on days 10, 20, 30, and 60 of the study. During the 30 days of continuous symptom monitoring, participants were also asked to wear a triaxial accelerometer to continuously record daytime activity and estimate evening sleep parameters. Of 285 participants recruited, 270 participants could be included in the study (gave full consent and successfully returned the study pack). In total, 91% (n = 246) of participants met the reporting criteria necessary to be defined as an engaged user over the 1-month study period (at least 15/30 days of symptoms and sleep diary and 2/4 follow-up questionnaires).

The success of these studies is due to the considered strategies used to maximize participant engagement, including focusing on usability of technology, functional ability of participants, and consideration of participant workload and time commitment; push or motivating factors, such as the use of reminders; data monitoring; provision of study contact details; and study support. This article describes the strategies used and lessons learned. It uses participant quotations (provided in participant e-mails, via social media, or in response to formal feedback requests) to highlight how successful the strategies were in promoting engagement.

## Patient advisory groups

First and foremost, it is important to consider the use of patient advisory groups (PAGs), who are well positioned to codesign the study by identifying potential barriers for participants and help craft possible solutions. Members of any PAG developed should comprise people who have lived experience with the condition or symptom of interest in the study. Ideally, participants have a range of levels of disease severity and experience with both technology and research more generally.

The authors’ PAGs comprised volunteers from the Greater Manchester, UK, area with chronic pain (Cloudy with a Chance of Pain) or rheumatoid arthritis (RA) (QUASAR), who responded to requests for participants disseminated through the University of Manchester Centre for Musculoskeletal Research User Group online support networks or social media. Participants had a range of experience with technology and included active smartphone users and individuals who had never used a smartphone. Most participants had some experience in research, although not specifically in research using digital data collection. PAG members were asked to discuss various aspects of study designs, including the frequency and content of data collection, and to identify any barriers or facilitators to participation in studies using digital data collection.

## Usability of technology—functional ability and workload and time required

### Functional Ability of the Study Population

Attrition is likely higher among people who experience functional/logistical limitations using the app. Thus, specific considerations must be given to the suitability of the devices provided for the target population. For example, the sleep monitor used with the QUASAR study has an event marker, which participants were asked to use to indicate when they got in and out of bed. The authors, however, had numerous reports from participants that they had difficulty with the monitor due to issues of dexterity and hand function:

I have put the monitor on this morning, and touched the centre disc I don’t have very good sensitivity in my fingers, so wonder if you can check your end that I am applying enough pressure.

—QUASAR participant

I have attempted to press the button…but I have no sense as to whether I am meant to feel the button depress…This may be because the fingers on my right hand are quite swollen and lack much feeling at the tips.

—QUASAR participant

In one instance, a participant came up with the solution to use a stylus used for their other devices and was able to successfully use the marker thereafter. Many participants stopped using the marker, instead focusing on completing the morning sleep diary to report the previous night’s sleep period. This issue was not noted within the QUASAR PAGs, which comprised patients with RA discussing aspects of the study design, or by the healthy volunteer pilot testers who wore the monitor for 7 days. The authors noted, however, that the monitor was extensively worn only by healthy volunteer testers and it may have been that this issue would have been highlighted if members of the PAGs had instead worn the monitor for the 7-day pilot study. Thus, despite the usefulness of the PAGs and piloting in identifying several key barriers and facilitators for participation in the study, these processes were not sufficient. In future, where a study requires the use of new or unfamiliar technology, it would be beneficial to conduct extensive patient piloting in the initial stages of study design and pilot test implementation.

### Workload and Time Required

Although some degree of attrition is inevitable in longitudinal research, it is likely that the attrition is greater and more rapid when participant burden (both in terms of frequency and complexity of data collection) is higher.^9^, ^17^ Yet, data collection for comprehensive mHealth studies must include the collection of data on all relevant exposures, outcomes, and putative confounders and may become particularly burdensome when all 3 variable types are time varying (ie, their values change over time.)^18^

It is essential that the study design considers the most parsimonious data collection protocol possible from a participant’s point of view, while being sufficiently comprehensive to collect all data necessary. The authors recommend that to optimize data collection, researchers should codesign their study with PAGs, thus gathering a range of opinions from individuals on the amount, frequency, timing, and type of data collection that can be best integrated into daily life. For example, the authors’ PAGs determined that it would be acceptable to report data at 1 time point per day for 6 months (latterly extended to 1 year [Cloudy with a Chance of Pain]) and twice per day for 1 month (QUASAR). In discussing the frequency of data collection per day, the authors were able to create data collection protocols, which participants found well suited to their lives:

It’s all very easy and straightforward to do every day.

—Cloudy with a Chance of Pain participant

It’s become a habit now, part of the routine of the day.

—Cloudy with a Chance of Pain participant

App is simple to use. Very convenient.

—QUASAR participant

Happy to partake in any studies that provide research into RA and don’t drastically disrupt my life!

—QUASAR participant

**Fig. 1:**
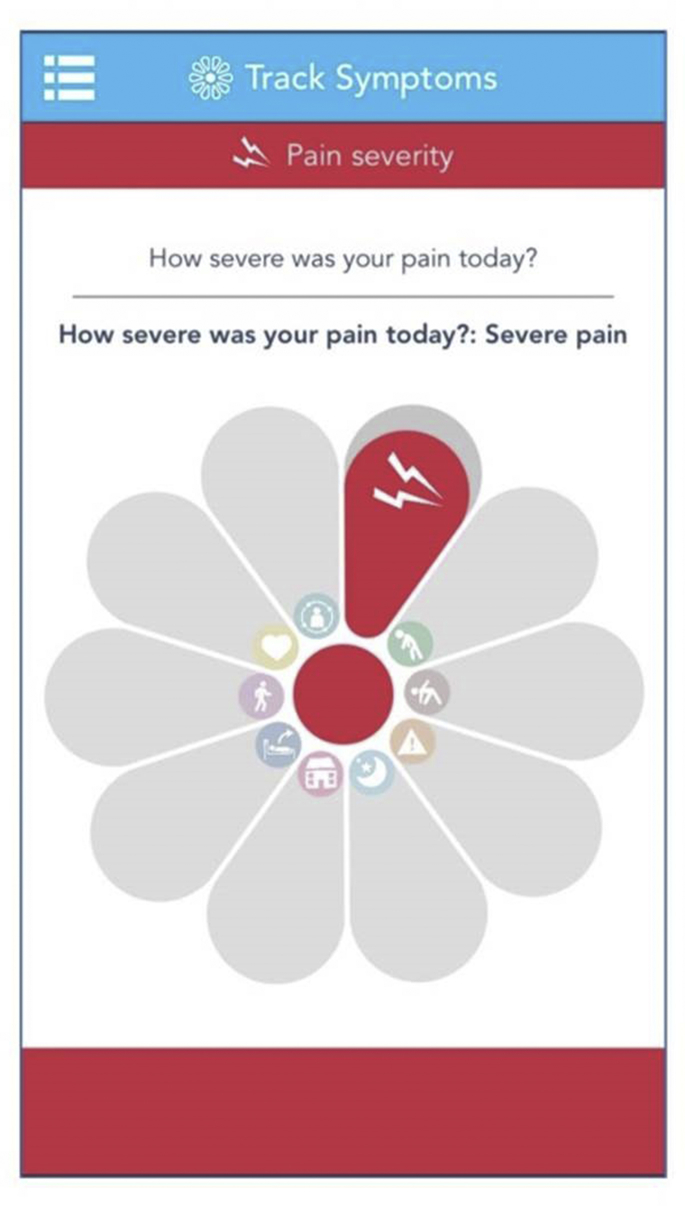
Main graphical interface used in Cloudy with a Chance of Pain and QUASAR. Each of the 10 segments represents a different symptom, such as pain severity (highlighted), measured on a 5-point ordinal scale.

The perceived ease of use is largely due to the core uMotif platform used across both studies that comprises a simple graphical interface (Fig. 1) and is attractive to users and quick to complete (estimated 1–2 minutes per entry).^6^, ^16^ The uMotif interface comprises 10 segments, each of which represents a symptom that can be moved to give an ordinal 5-point outcome (eg, pain severity measured as 1 “no pain” to 5 “very severe pain”). Participants tap each of the 10 segments to highlight the relevant symptom and move the colored section to denote symptom severity.

Participant burden may be reduced further by the use of passive monitoring. Passive monitoring is defined as a data collection technique that can collect relevant information without active engagement from the participant. Techniques may include the use of physical activity monitors (eg, accelerometers), heart rate or blood pressure monitors, or other built-in features of the mHealth device, such as gyroscopes.^6^, ^19^, ^20^, ^21^ In the authors’ studies, the built-in GPS tracking on participants’ smartphones (Cloudy with a Chance of Pain) and sleep monitors (QUASAR) was used to capture data passively. The authors have found that such methods of data collection are acceptable to participants providing they do not experience a reduction in the battery life of their devices (eg, GPS) or that any wearable technology (eg, sleep monitors) is discreet and unobtrusive. No privacy issues were raised with respect to the collection of geolocation, although it is not known whether any potential participants were put off of participating in the Cloudy with a Chance of Pain study due to concerns of privacy.

Embedding passive monitoring within studies not only may enable improved engagement and reduced participant burden but also may give greater dimensionality to the data collected if used to complement subjective assessments, such as when measuring sleep.^6^ This increased dimensionality may serve to improve the accuracy of assessments, if objective markers can replace commonly used subjective assessments, which may be subject to reporting errors and biases. Although promising, there remains a need to validate and standardize many of the objective outputs available.

## Push factors

Push factors to promote engagement may range from generic strategies, such as the use of automatic daily prompts or alerts for data completion, to a more intensive and bespoke process of real-time data monitoring and targeted completion reminders. Other factors that may push participants to engage may include ongoing study feedback, networking effects, and opportunities to interact with other participants within study communities.^9^ The decision regarding how many different types of push factors to use, however, is a balance between how labor intensive the processes are for software developers to create, or the study team to deliver, and the benefits received.

### Automated Reminders

Automated reminders and notifications typically are built-in features of mHealth studies and increase the chances of collecting the data required, because data entry not only is reliant on a participant’s memory but also is prompted. Automated reminders are particularly beneficial because minimal, or no, input is required on the part of the participant or researcher to set up and receive the reminders. Reminders may be delivered at fixed at times each day or semirandomly throughout the day.^19^ Within the authors’ studies, reminders were discussed within the relevant PAGs and agreed suitable reminder times were fixed at either 6.24 pm (Cloudy with a Chance of Pain) or 8.00 am and 6.00 pm (QUASAR). It is necessary to use caution, however, in deciding the timeframe in which reminders are sent because certain times may be unsuitable for specific participants, such as those who are employed in shift work (who were necessarily excluded from the QUASAR study) or those who have a fixed social routine:

I have quite a busy life and go swimming early most mornings then sometimes I forget if I have entered my symptoms.

—QUASAR participant

Is it possible to change the times for the reminders? Now I've done it a couple of times I realise they're not quite right for me at the moment. I'd ideally like them at 7:30 and 18:30.

—QUASAR participant

For future studies, it may be worth considering whether it would be beneficial to allow participants to dictate the times at which they would like to receive their reminders, provided this flexibility has no detrimental impact on the exposures and outcomes under investigation (ie, neither factor is time-sensitive).

Real-time data monitoring and targeted completion reminders

If reminders are unsuccessful and participants have not completed data collection, it has been shown that real-time data monitoring and active chasing of participants (ie, sending targeted completion reminders) can be successful in preventing dropout and maximizing data completion.^9^ This approach was adopted within the QUASAR study, with a data monitoring process was developed to identify participants at risk of disengagement and to send participants 1 of 3 noncompletion text messages, to encourage re-engagement and data completion.

**Fig. 2:**
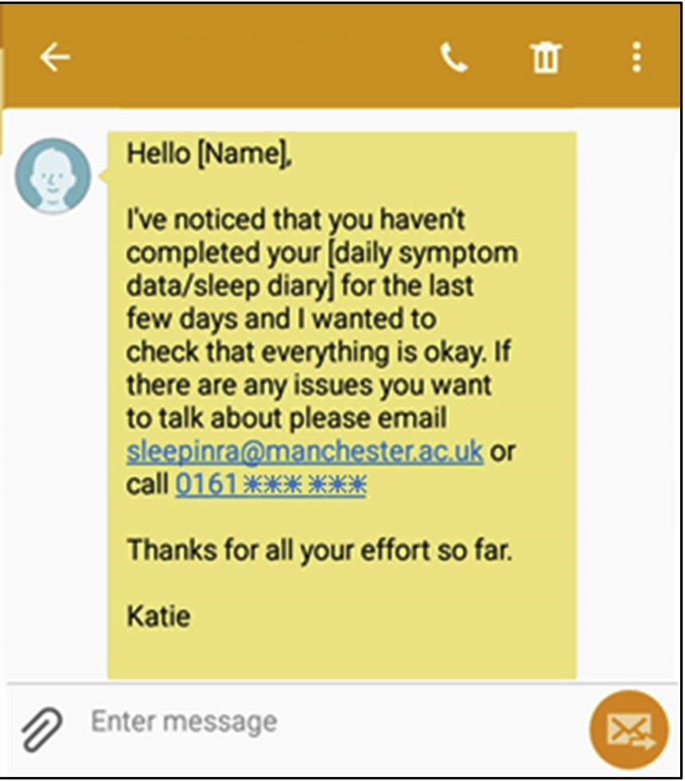
Example of noncompletion reminder text message sent during the QUASAR study.

Data monitoring was completed every second business day (Monday–Friday) after the manual download of data from the study portal created by uMotif to the research team data environment. An internally developed database script then analyzed the data download and alerted the study team (via e-mail) to any participants who had not completed symptom or sleep diary data for 3 days or who had not completed a follow-up as expected. On receiving the alert, the study team checked the report and confirmed that relevant participants should receive a text message to encourage completion (eg, Fig. 2). The study team could ensure that participants were not continually chased for data completion; instead, a maximum of 2 messages would be sent before a phone call was made to try to directly contact the participant and discuss any problems.

**Fig. 3:**
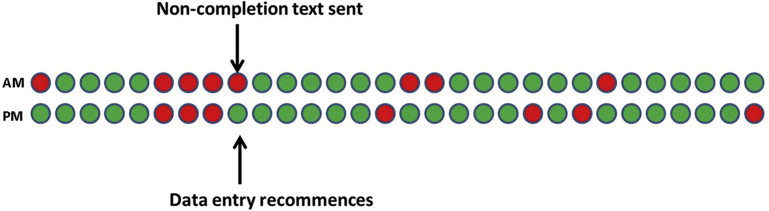
Data-entry report for the QUASAR study, with notation for when noncompletion text was sent and data entry recommenced.

A total of 315 noncompletion messages were sent during the QUASAR study to 181 (63.5%) unique patients: 7 registrations not completed on the agreed study start day, 52 greater than or equal to 3 days of missing symptom or sleep diary data (14 symptoms and 38 sleep diaries), and 256 follow-up questionnaires not completed on the day expected. After these text messages, 3 registrations were completed (42%), 201 follow-up questionnaires were completed (79%), and 36 people recommenced frequent completion of sleep diary or symptom data (69%; example shown in Fig. 3). With the exception of the follow-up questionnaires, few people required repeated chasing to complete data entry. Just 3 people (1%) were contacted twice to prompt completion of sleep diaries or symptoms, whereas 49 people were contacted twice or more to complete follow-up questionnaires (eg, they had not completed their days 10 and 20 follow-ups). A total of 3 people (1%) were contacted for missingness of symptoms, sleep diaries, and at least 1 follow-up.

The application of such near real-time screening for data missingness is clearly beneficial, but, unless processes of data monitoring and text message creation are automated, the use of such strategies may be labor intensive for the research team. Thus, it is important to balance the effort likely to be expended with the achievable benefits (eg, the increase in amount of completed data or increased proportion of people meeting minimum data requirement). Rules also should be developed to agree to a reasonable time period, or frequency, with which participants can be prompted to provide data, without risking participants feeling harassed or coerced into providing data.

### Personal Motivations

Individuals may be more likely to participate in studies of experiences that have affected them personally or in studies where they perceive a wider societal benefit.^22^ Personal motivations for participating in studies were highlighted in PAGs held for both exemplar studies as being the desire to contribute to answering an understandable and engaging research question (Cloudy with a Chance of Pain) and due to personal experience of the poor management of an illness or symptom and desire to develop a suitable management solution (QUASAR). Importantly, by addressing a research question that is important or meaningful to the participants, their contribution is perceived of greater personal and societal benefit, thereby increasing the likelihood of engagement:

It’s really good to see some in-depth research into sleep and RA… sleep is way more important than most of us give it credit for, and RA is no doubt extremely disruptive to it.

—QUASAR participant

I personally am in no doubt the weather/pressure/temperature has a monumental effect on conditions of chronic pain and am very grateful that this study is being done, and that someone actually wants to research it and help

—Cloudy with a Chance of Pain participant

**Fig. 4:**
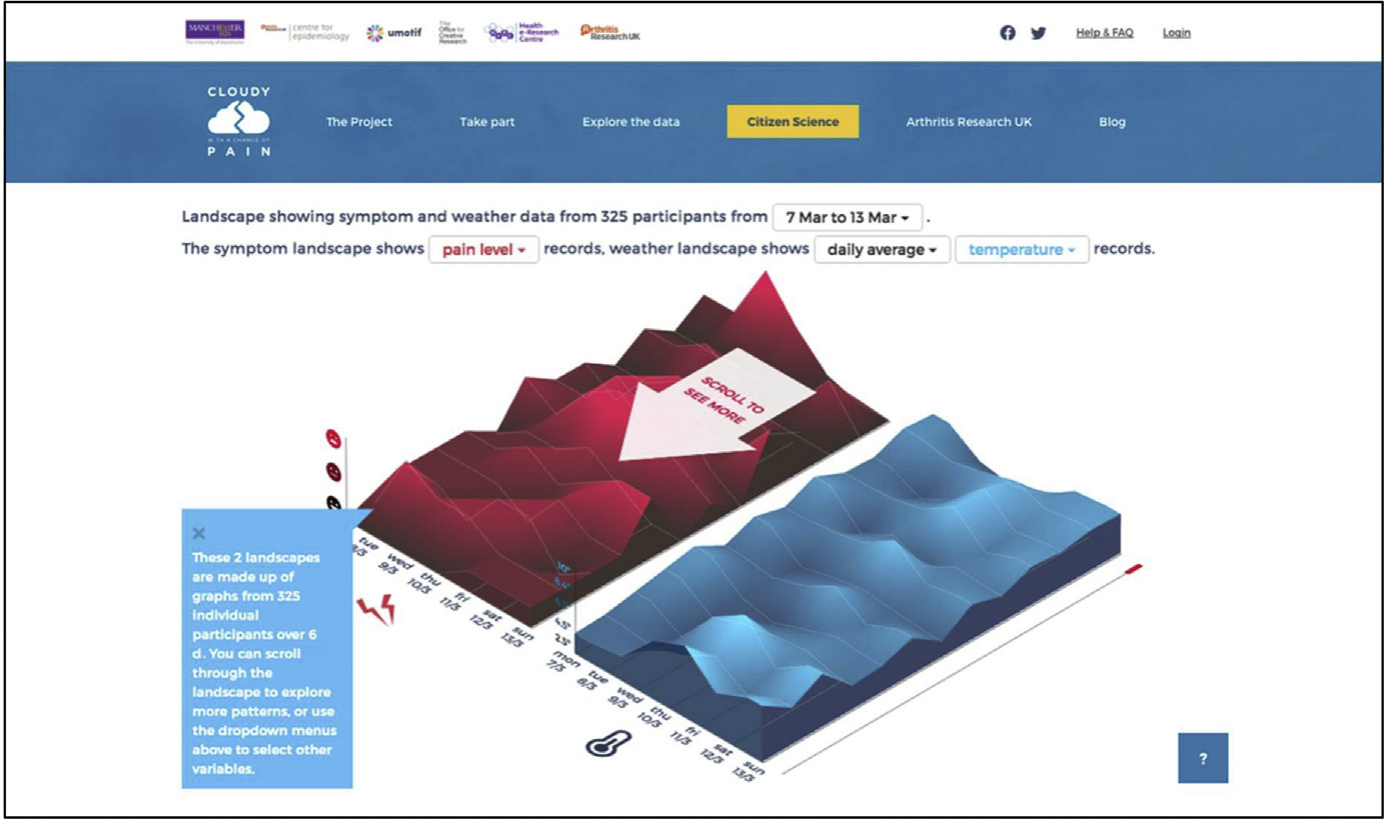
Screenshot of the citizen science project for Cloudy with a Chance of Pain.

It is noteworthy that Cloudy with a Chance of Pain built on participants’ desires to contribute to answering the research question by asking participants and the wider public to engage in a citizen science project (Fig. 4) to view and interact with the data collected during the study and submit new, or revised, hypotheses about the link between pain and the weather. A total of 418 hypotheses were submitted by both study participants and members of the wider public. Participants were also reminded that their ongoing data completion and engagement were valued in text messages (QUASAR), weekly newsletters (Cloudy with a Chance of Pain), and relevant study emails. As a result, participants were able to constantly see the benefits of ongoing data completion and were aware that their data are valuable and actively contributing to the researcher’s ability to answer the studies aim.

### Study Feedback

**Fig. 5:**
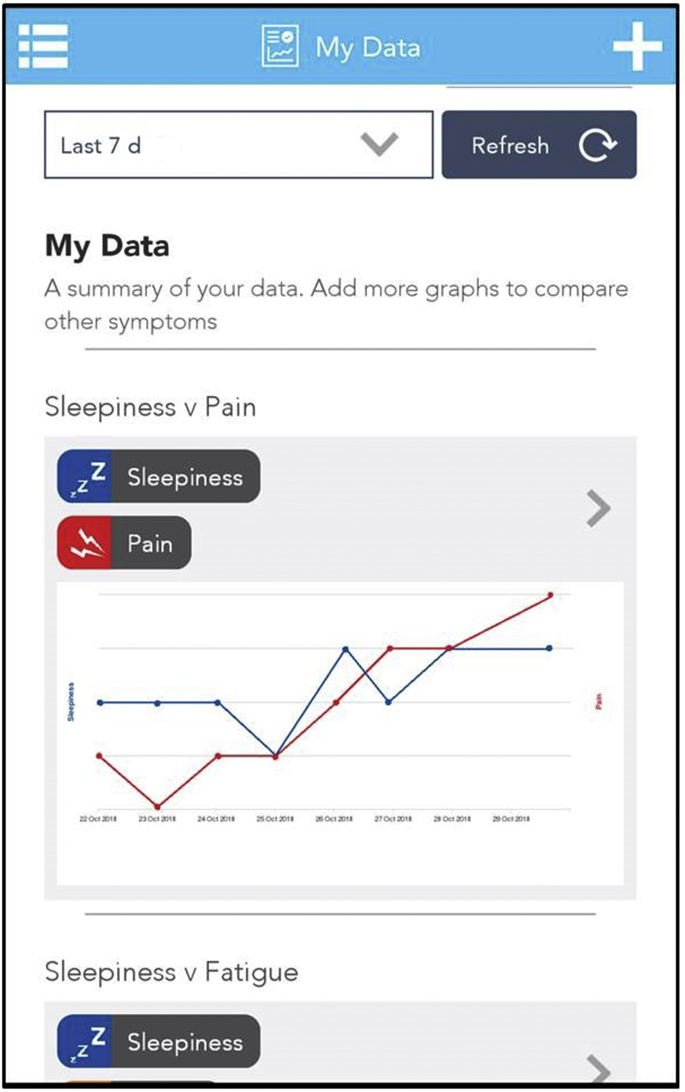
Example participant feedback provided via the symptom tracking feature of the uMotif app.

In addition to a desire to contribute data to address the research question, PAG members highlighted that participants may wish to personally benefit from taking part in studies by receiving feedback on (personal) study results. In response to this, feedback has been provided to participants in a variety of ways. First, throughout the studies, participants could also see their own symptom data in the app (Fig. 5). In doing so, participants were able to constantly see the benefits of ongoing data completion in enabling them to track their own condition, to identify triggers of symptom flares or decline in health, and to improve communication with friends, family, and health care professionals^17^:

Making the observations for this study has made me aware of how much better I feel if I spend a reasonable amount of time out of doors, and moderately active.

—Cloudy with a Chance of Pain participant

Right from the start I learnt to change my sleeping habits. This has been an absolute life changer in coping with my pain.

—QUASAR participant

When would the results be available? Be good to get my doctors to look at them.

—QUASAR participant

I am finding the graph data fascinating. Plus it's great to have a chance to chart my illness in so many ways, giving new info to my rheumatology consultant.

—Cloudy with a Chance of Pain participant

**Fig. 6:**
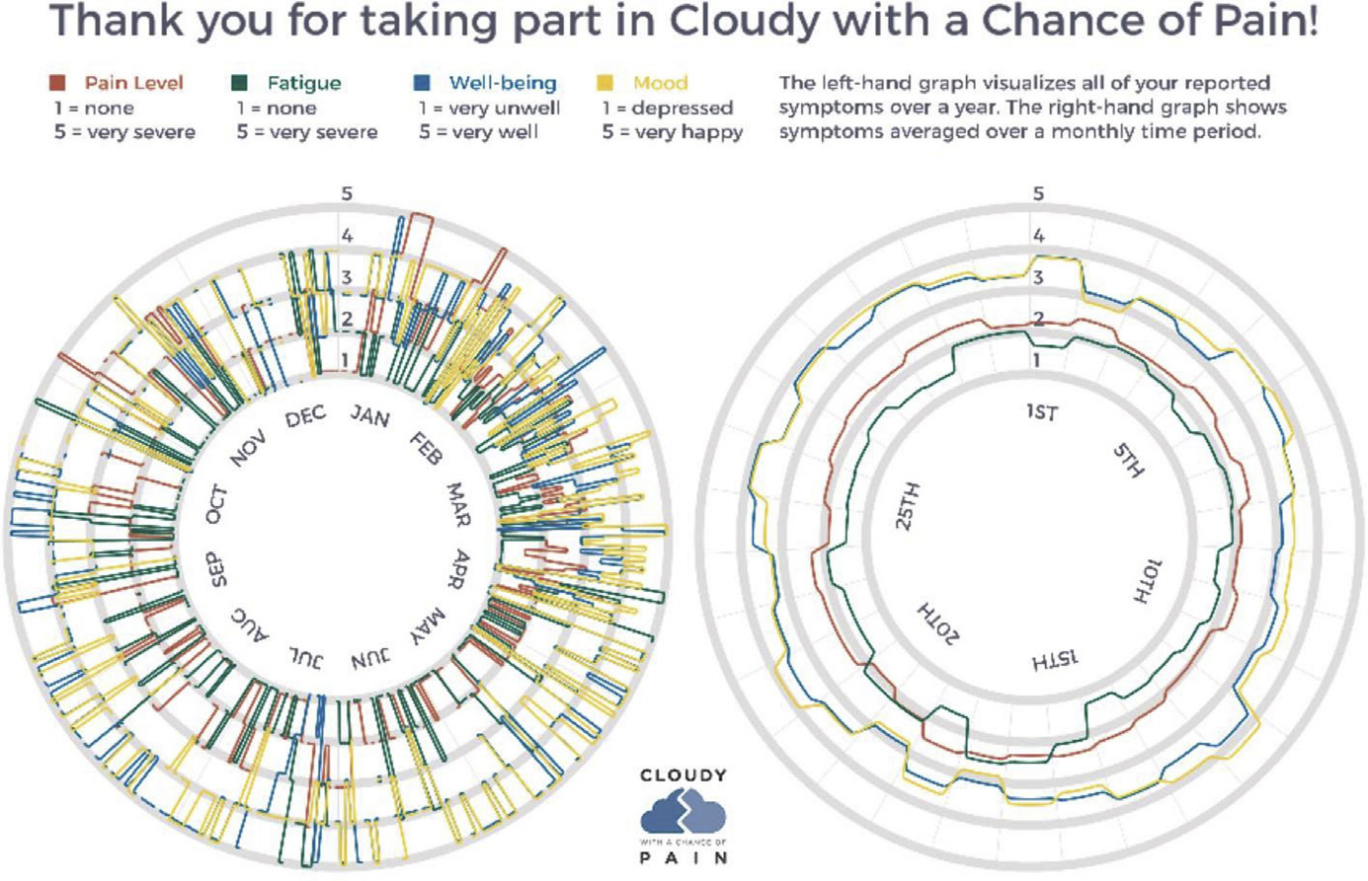
Example participant feedback postcard provided in Cloudy with a Chance of Pain.

It is vital to highlight that several participants in the PAG for QUASAR and many of the participants recruited to the study emphasized negative experiences from previous studies, which had promised feedback but had failed to provide it. In such cases, participants mentioned that they had been put off taking part in research because it felt like they received no benefits to participation. Mindful that comprehensive analysis can take many months, it is worth considering providing participants with interim study results. With this in mind, feedback was also provided to participants more formally within personalized end-of-study reports. In QUASAR, participants were provided with an end-of-study report that detailed their total hours of sleep each night for 1 week and their corresponding average pain, fatigue, and well-being scores. In Cloudy with a Chance of Pain, participants were invited to request a bespoke study postcard of their data, designed in collaboration with a creative researcher, which showed participants all of their symptoms reported throughout the 12-month study period (Fig. 6 [*left*]) and the average of those symptoms over a monthly time period (see Fig. 6 [*right*]).

### Networking Effects and Community Building

The significance of creating a study community was highlighted within the Cloudy with a Chance of Pain study, in which various (optional) social media and support channels were also made available to participants to engage with the study team and other participants.

In the first instance, an online community (786 members and 107 posts) was established by the study team and hosted by HealthUnlocked, a UK patient support network. Within this community, participants (who may have been preexisting members of HealthUnlocked) could discuss their study experience and connect with other participants, to discuss their health more widely. There were no restrictions on what participants could discuss within the community, provided they followed HealthUnlocked’s terms of use (https://healthunlocked.com/policies/terms).

Furthermore, participants were able to connect with the research team and other participants via the study team’s presence on social media, including Twitter (@CloudyPain; 883 followers), Facebook (Cloudy with a Chance of Pain; 585 likes), and Instagram (@Cloudy_Pain; 49 followers). Finally, weekly newsletters and an online blog (https://www.cloudywithachanceofpain.com/blog) disseminated information about study progress and included guest articles from participants, charity partners, researchers, and funders. Importantly, by establishing the study community, it was possible to enable participants to feel empowered to share their experience.

After the end of the study, all social media accounts remained live to promote dissemination of the final results of the study and any relevant interim findings. The community page on HealthUnlocked also remained live for participants to continue using at their discretion, but it was not actively monitored by the study team after the end of the study.

## Personal contact and ability to obtain help

In addition to the support provided by study communities, personal, as opposed to virtual, contact was highlighted as an essential provision for mHealth studies. In particular, QUASAR PAG members believed that having personal contact was important to make participants feel valued and more likely to complete the data collection protocol. Specifically, PAG members recognized that many people may never have participated in mHealth studies before, and there was a need for ongoing reassurance and feeling that their participation is important. As a result, PAG members requested the development of processes to ensure participants had access to ongoing study support by phone or e-mail, courtesy calls, or check-ins during the study and ongoing feedback or study progress updates.

**Fig. 7:**
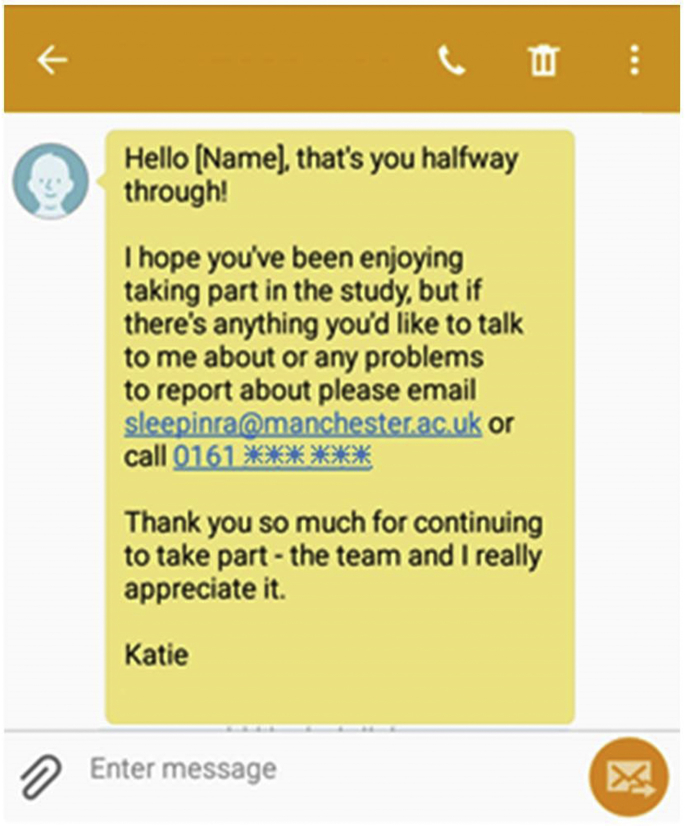
Example motivational text message sent during the QUASAR study.

Although the experience of personal contact is preferred by participants, the decision to provide of courtesy calls or check-ins must be made in consideration of the study resources and the sample size to be recruited. For example, without substantial logistical support, it would have been impossible to provide courtesy calls to each of the 13,256 people recruited to Cloudy with a Chance of Pain. Due to staff capacity and study support, neither was it feasible for such calls to be made in the QUASAR study, but instead a decision was made to adopt the use of personalized motivational messages (ie, referring to the participant by name [Fig. 7]), which could be automatically generated and sent by a computer program but could offer support and opportunities for participants to be telephoned by a member of the study team. Any contact requests were responded to within 24 hours, except in exceptional circumstances. In addition to e-mail support, participants were able to telephone both study teams at any stage in the study.

Participants were free to contact members of the study team to discuss any issues they wanted to, including concerns they had about the study or any technical difficulties they were experiencing. The only restriction was that no medical advice would be provided over the phone. Instances where participants contacted the study team tended to be to gain support for one-off issues, such as difficulty registering, reporting an inability to use the event marker on the sleep monitor, or to indicate their withdrawal from the study. A small group of people (n <20) made repeat contacts to the study team to discuss issues experienced throughout the study, including problems with data entry or study reminders, or to check that they were completing data correctly.

In addition to study-specific support, technical support was provided by the app providers, uMotif, via a designated help desk. A mutual sharing of relevant emails between the help desk and study team members ensured that any misdirected emails could still be responded to immediately (eg, requests for help with the sleep monitor sent to the uMotif help desk, instead of the QUASAR study team). As with the main study email account, help requests to uMotif were responded to within 24 hours to 48 hours, except in exceptional circumstances.

Investment in ongoing study support is integral to the success of future studies, because participants specifically identified the ability to obtain help as a factor for ongoing participation. In particular, within the QUASAR study, participants principally had 1 point of contact during the study, with the study coordinator responsible for all recruitment calls and participant contacts, and participants specifically highlighted this continuity of communication as a benefit of the study. Thea authors recommend such an approach be adopted, if feasible, within future mHealth studies.

## Summary

It is well recognized that mHealth technologies provide an exciting opportunity for researchers to obtain frequent and repeated measures for a range of self-report data and objective assessments and address hitherto unanswerable questions. Despite these benefits, mHealth studies are vulnerable to high attrition rates and it is essential that researchers actively consider strategies to maximize participant engagement. In 2 successful mHealth studies, the authors focused on factors associated with usability of technology, including functional ability of participants and consideration of participant workload and time commitment; push or motivating factors, such as the use of reminders and data monitoring; and the provision of personal contact and study support. Future studies should consider adopting and advancing these approaches at an early stage of study design to maximize engagement.
